# COVID-19, curtailed clerkships, and competency: Making graduation decisions in the midst of a global pandemic

**DOI:** 10.36834/cmej.70432

**Published:** 2020-12-07

**Authors:** Janeve Desy, Adrian Harvey, Kevin Busche, Sarah Weeks, Michael Paget, Christopher Naugler, Lisa Welikovitch, Kevin McLaughlin

**Affiliations:** 1 Office of Undergraduate Medical Education, Cumming School of Medicine, University of Calgary, Alberta, Canada.; 2 Office of Post-graduate Medical Education, Cumming School of Medicine, University of Calgary, Alberta, Canada.

*“Make the best use of what is in your power, and take the rest as it happens.” —* Epictetus

In December 2019, reports emerged from Wuhan in Hubei province, China, of infection caused by severe acute respiratory syndrome coronavirus 2 (SARS-CoV-2) that we now typically refer to as Coronavirus disease 2019, or COVID-19. Within three months, spread of this virus around the world has created chaos and uncertainty in almost every aspect of our lives. Included within the uncertainty is the fate of students from the Class of 2020. In the interests of physical distancing, we were forced to curtail their clerkship training, to convert clinical rotations to on-line learning experiences, and to accept that outstanding workplace assessments won’t arrive on time, if at all. Through no fault of their own, this cohort of students was being prevented from completing the in-person learning experiences and assessments that hitherto have been required in order to graduate to residency. Also holding their breath are the postgraduate program directors and patient who rely upon us to graduate students who are ready for residency training (and remediate those who are not). So, how should we make graduation decisions based on incomplete information due to a global pandemic?

In this article, we will argue that despite the curtailment of clerkship training and missing data on assessment, the greater good is served by graduating students for whom there is *clear and convincing evidence* of readiness for reactive^1^ supervision when performing the core Entrustable Professional Activities (EPAs) of graduating medical students.^1-4^ We will then discuss the challenges of making graduation decisions during an evolving pandemic, including why decision-making in this context may be particularly susceptible to bias. We will finish by describing how we took the opportunity offered by this pandemic to accelerate our transition to Competency-Based Medical Education (CBME) in order to create a graduation decision-making process that allowed us to make decisions that we felt were appropriate and defensible even during these challenging times.^5,6^

## To graduate or not to graduate, that is the question

When the COVID-19 pandemic struck North America, most UME programs were still in the CBME planning stages and, rather than making graduation decisions based upon performance of EPAs, each had their own unique criteria for graduation that typically included the completion of mandatory learning experiences, a minimum time period of training, and performance at or above “the expected level” on all knowledge, clinical skills, and workplace-based assessments. Like most, we were not prepared for this pandemic and had not anticipated a situation where we might be required to make graduation decisions with *missing data*. So, do curtailed clerkships and missing data imply that the class of 2020 will not be as prepared for residency training as their predecessors? And if we graduate this cohort, will this inflict further suffering on post-graduate program directors and patients in the form of substandard care from under-prepared graduates?

Unfortunately, we have no idea when the current pandemic will end. We must also anticipate a second wave (or multiple waves) following relaxation of physical distancing, which may result in further interruptions of clerkship training. And, alongside these concerns is the fact that postgraduate training has largely continued through the COVID-19 pandemic – so first year residents will soon become second year residents and graduating medical students will be needed to fill the training/service void. Thus, those of us involved in UME are faced with a difficult dilemma: do we maintain our previously immutable criteria for graduation and delay graduation of our medical students for the foreseeable future, or do we adjust our criteria for graduation?

For students from the Class of 2020 at the Cumming School of Medicine, we decided to adjust our criteria for graduation rather than delay graduation indefinitely. Specifically, we decided to accelerate our transition from our traditional graduation requirements that included completion of all clinical rotations with ratings at or above expected level on all summative assessments to a CBME assessment format where we recommend graduation of students deemed ready for reactive supervision on the 12 core EPAs of graduating medical students in Canada.^3,7^ Given our fiduciary responsibility to patients, this was a decision that was made after much deliberation and ultimately based upon five points:

### 1.Length of training is not a reliable surrogate of readiness for reactive supervision

Sufficient clerkship training is clearly a prerequisite for graduating to residency, but the fact that long-term performance is similar in graduates from three-year and four-year programs suggests that the duration of clerkship training does not in itself predict readiness for reactive supervision.^8^ As proponents of CBME have long argued, it seems irrational to base graduation decision on the assumption that all students require proactive supervision until the final day of clerkship training when most, if not all, are then suddenly ready for reactive supervision.^9^ Thus, we felt that delaying graduation of students due their failure to complete our pre-set duration of in-person training was not justified.

### 2,We already had most of the data needed to determine readiness for reactive supervision

A commonly referenced guideline regarding missing data is that loss of less than 5% likely results in little bias, whereas loss of more than 20% should cause us to question the validity of our findings.^10,11^ At the Cumming School of Medicine, where we have a three-year curriculum, our students had completed more than 90% of their scheduled clerkship learning experience when their training was interrupted.^12^ So, while accepting that there may still be validity issues when there is less than 10% missing data, based upon the parameters of this guideline, most would accept the validity argument for graduation decisions based upon more than 90% of a complete dataset. Had the COVID-19 pandemic arrived in North America half way through clerkship training then we could not justify graduating the Class of 2020 on time.

### 3.We already had EPA-based assessment data

Descriptive anchors for rating scales used to assess clinical and workplace performance of students typically categorize *overall* performance as unsatisfactory/satisfactory/outstanding or below/at/above an ill-defined “expected level.” By contrast, CBME articulates performance in terms of *recommended level of supervision* when performing *specific* EPAs.^1,2,13^ While the traditional assessment tools used in UME to assess clinical performance, including objective structured clinical examinations (OSCE) and in-training evaluation reports (ITERs), can be adapted to assess EPAs, there is no valid method for switching between outcomes of “overall performance” and “recommended level of supervision while performing a specific EPA.” Fortunately, for the Class of 2020 we had an EPA-based clerkship OSCE, our ITER items were mapped to the core EPAs, and we had already started to ask a subset of clinical supervisors to rate students as requiring proactive versus reactive supervision on a clinical rotation in addition to rating their overall performance.^7^

### 4.Most of the students had already matched to residency positions

From the perspective of medical students, the most important outcome of their undergraduate training is likely the degree to which they are successful in the residency matching process.^14,15^ Imagine, therefore, the scenario where the pandemic also interrupts this. Allowing students to start residency on time may have required interviews to be completed via videoconference, or applicants and programs could have made choices based upon prior encounters, file review, or even a lottery matching system. While graduating the Class of 2020 has been challenging, if COVID-19 had arrived two months earlier so that compromised career choice added to the chaos of curtailed clerkships, timely graduation would probably have been inconceivable.

### 5.Distributive justice is served by graduating students who are typically ready

Having decided that we could make a validity argument for our graduation decisions, we then had to decide if it was *fair* to graduate students from the Class of 2020. Distributive justice refers to fairness in the distribution of outcomes, both rewards and costs, between different stakeholders or group members.^16,17^ We felt that delaying the start of residency for all students – most of whom are ready for reactive supervision – would be unfair not only to those students, but also to the residency programs who selected them and the healthcare system that relies upon the influx of new graduates. Thus, graduating students who are ready for reactive supervision appears to serve distributive justice since all stakeholders are more likely to benefit from this rather than an indefinite delay in graduation.

It is important to emphasize that distributive justice is not served by simply graduating all students from the Class of 2020. Distributive justice is only served by making appropriate graduation decision, i.e., to graduate only students who are ready for reactive supervision and not those who still require proactive supervision. While this may seem like an obvious statement, recent literature suggests that graduating students who are ill-equipped for residency training is not a rare event, and we should also be concerned that the likelihood of inappropriate graduation decisions may increase in the midst of a pandemic.^18,19^

## Why graduation decisions may be more prone to bias during a pandemic

A recent article by Santen and colleagues explores the sensitive topic of medical schools graduating rather than remediating students with academic or non-academic difficulties. Failure-to-fail is a well-recognized issue in clinical assessment and, while this can be partly attributed to the challenge of assessing ill-defined constructs with imprecise tools, systematic rater biases may also play a role.^19,20^

When a teacher evaluates a student there is the inherent potential for bias, such as *anchoring* and the closely related *confirmation bias*, and if the teacher has a favourable impression of the student then these biases may induce leniency in the rating of the student’s performance.^21,22^ And, when this teacher is also part of the undergraduate training program, additional biases may further compound decision-making. For example, having compassion for students and anticipating the negative psychological and financial impacts of delayed graduation could introduce *impact bias* that reduces the likelihood of recommending remediation (or dismissal).^23^ The probability of remediation may be decreased further by *self-serving bias* if those making this decision anticipate potentially negative consequences to themselves and their training program from their decision to recommend remediation rather than graduation.^24^ If these biases influence graduation decision during “normal” times, how might their effect change during a pandemic? Fuelled by the fact that, through no fault of their own, medical students have been prevented from completing outstanding and remedial clinical rotations and post-graduate program directors from preparing their July on-call schedules, will impact bias spread rapidly throughout UME? And might there be an outbreak of self-serving bias among those of us involved in organizing the undergraduate curriculum as we anticipate a potential “surge” of medical students (both the class of 2020 and 2021) scrambling for limited spots on clinical rotations (and potentially future residency match cycles) beginning on a yet-to-be determined date that is conditional upon “flattening of the curve”?

## How to make difficult decisions during difficult times

In the face of interrupted training and missing data due to the COVID-19 global pandemic, we decided that the most justifiable way to make graduation decisions for the Class of 2020 was to change our criteria for graduation by accelerating our transition towards CBME. However, we did this mindful of the fact that we would be required to make this transition more rapidly than originally planned, and would do so at a time when the risk of bias in graduation decisions may be increased. Faced with these challenges, we began by defining our new criteria for graduation: “for a student to graduate to residency training, this must be the consensus recommendation of a disparate group of individuals who, having considered all of the available data, believe that this student is ready for reactive supervision when performing each of the core EPAs of a graduating medical student.” Based upon this description, we then identified the key components of our process for making graduation decisions:

### 1.Our graduation decisions should be based upon the Wisdom of the Crowd

Readiness for reactive supervision (or *entrustability* or *competence*) is a difficult-to-define construct that includes various attributes of a student, such as ability, reliability, integrity, and humility, and the subjective assessments of multiple raters on whether these attributes are sufficiently developed to allow for success at the next stage of their training.^13^ This construct cannot be adequately described by a single number on a Likert scale or a multiple-choice question (MCQ) examination score alone, or by a mathematical formula.^25^ Instead, data on readiness is provided by a variety of assessment tools that describe performance using different combinations of numbers and words. And, since all of these tools are at least partially subjective and assess performance in different content areas at different times during the student’s training, data on the outcome of readiness may be inconsistent. At a societal level, when we are required to make high-stakes decisions based upon inconsistent and subjective data, the usual procedure is to ask a jury to make a recommendation based upon all of the available data and to then accept the *Wisdom of the Crowd*, so we adopted this model for our graduation decisions.^26^

### 2.Our Crowd should include stakeholders who are not student advocates

The potential for conflict-of-interest is inherent within the medical educator role as we strive for balance in our responsibilities to our students, colleagues, and patients. Those of us involved in undergraduate training realize that a decision to graduate a student who is not ready for proactive supervision may ultimately have a negative impact on patients, those who participate in post-graduate training, and the medical profession in general, but it is part of the human condition to choose short-term self-reward over long-term negative consequences to others.^27^ Rather than being a deliberate act, rater bias is typically subconscious, so when we ask medical educators to advocate for students they will typically do just that – and anchoring, confirmation bias, impact and self-serving biases may all be concealed within this “advocacy”. Thus, in order to achieve balance in the Crowd – and to reduce the risk of additional biases that can affect collective decision-making, such as *groupthink* and *in-group bias* – the jury that makes graduation decisions should include individuals representing stakeholders who would face the consequences of inappropriate graduation decisions, including those involved in post-graduate training and from society in general.^28,29^

### 3.Our criteria for graduation should be unconditional

As medical educators, we naturally have compassion for students from the Class of 2020 that have been adversely affected by the COVID-19 pandemic. Students from this class who are ready for reactive supervision deserve to be graduated to residency, and we also owe it to our post-graduate colleagues to have them delivered on time. However, for all involved in medicine and medical training, our overriding and fiduciary responsibility is to patients. Thus, irrespective of the context or student cohort, those participating in graduation decisions are behooved to maintain the standard of performance that is required for graduation, which is readiness for reactive supervision.^3,4^

### 4.Our decision on readiness should be based upon “clear and convincing evidence”

When using a jury approach to decision-making, we need to establish a “standard of proof.” In Canadian law, the standards of proof most frequently used are “beyond reasonable doubt” (for criminal cases) and “balance of probabilities” (for civil cases).^30^ Unfortunately, neither of these seems appropriate for setting a standard of performance for graduation to residency as the former would require close to consistent and flawless performance, while slightly more than half of ratings at ready for reactive supervision would suffice for the latter. Assessments in medical school rarely have the minimum performance level set at 99% or 50.1%. In US law, there is an alternative standard of proof, referred to as “clear and convincing evidence.”^31^ This standard is positioned between beyond reasonable doubt and balance of probabilities, and appears to be the standard of proof that is most consistent with the process of setting a minimum performance level in medical education, including CBME.^1^

### 5.Our graduation decisions should be achieved by consensus

**Figure 1:**
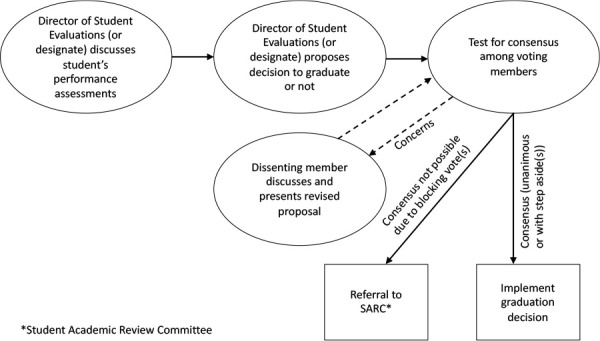
Revised process for making graduation decisions at the Cumming School of Medicine, University of Calgary

When multiple stakeholders contribute to decision-making, those in the minority can be consistently overruled by majority decision-making. Thus, for our graduation decisions, we opted for a consensus decision-making process with a decision rule based upon unanimity.^32^ This process begins by the Director of Student Evaluations discussing the performance of each student and then making a proposal to graduate or not based upon the student’s perceived readiness for reactive supervision. In order to mitigate risk of bias that may arise from prior knowledge of the student (discussed above) – or biases, such as stereotyping that could arise from simply knowing demographic information – all students are referred to using a unique identification number rather than using their name, student identification number, or a gender pronoun.^33^ Following the proposal, we test for consensus and, if achieved, this decision is then implemented. Where consensus is not achieved, each dissenting member discusses their concerns and presents a revised proposal. This process continues until there is either consensus among all voting members or dissenting members step aside in order for a proposal to be passed and implemented. If one or more voting members blocks a proposal then the graduation decision for this student is referred to the Student Academic Review Committee (Figure 1).

## Assessing the impact of COVID on readiness

When we make changes that are well thought out and planned in advance, we should never assume that our new process will be equivalent to or better than its predecessor – and we should certainly not assume this when rapid change is enforced by a pandemic. Similarly, articulating biases that might impact graduation decisions is not a vaccine that makes us immune to these. Graduation decision for the Class of 2020 might merit a question mark or an asterix, although we would argue that previous graduation decisions were also not bonded. For the past 17 years we have asked post-graduate program directors to provide feedback on the performance on all of our graduates during their first year of residency, and for the past three years this has been in the form of an EPA-based assessment tool that gathers both qualitative and quantitative data.^34^ This tool will allow us to prospectively compare performance of our graduates before and after our transition to jury-based graduation decisions and also during the COVID-19 pandemic.

## Conclusion

The current COVID-19 pandemic has created a healthcare crisis and severely impacted undergraduate medical training. Yet, it has also forced us to question our current processes and provided us an opportunity for change. We have tried to take advantage of this opportunity by introducing a graduation decision-making process that is consistent with CBME and, more importantly, is more flexible than our previous process. As Sir Winston Churchill famously advised, we should “never let a good crisis go to waste.”

## Footnotes

1 On the Entrustment scale, the recommended level of supervision for residents is “May act under reactive supervision” compared to “May act under proactive, ongoing, full supervision” for clerks.^7^
